# Impact of COVID‐19 on inpatient referral of acute heart failure: a single‐centre experience from the south‐west of the UK

**DOI:** 10.1002/ehf2.13158

**Published:** 2021-01-06

**Authors:** Gemina Doolub, Chih Wong, Lynsey Hewitson, Ahmed Mohamed, Fraser Todd, Laisha Gogola, Andrew Skyrme‐Jones, Shahid Aziz, Eva Sammut, Amardeep Dastidar

**Affiliations:** ^1^ North Bristol NHS Trust Bristol UK; ^2^ University Hospitals Bristol and Weston NHS Foundation Trust Bristol UK; ^3^ University of Bristol Bristol UK

**Keywords:** Acute heart failure, COVID‐19, Mortality

## Abstract

**Aims:**

Healthcare services worldwide have been significantly impacted by the COVID‐19 pandemic. Recent reports have shown a decline in hospitalization for emergency cardiac conditions. The impact of the COVID‐19 pandemic on hospitalization and particularly mortality due to acute heart failure has not been thoroughly described.

**Methods and results:**

In this single‐centre observational study, we examined referrals to the acute heart failure team over a period of 16 weeks (7 January to 27 April 2020) spanning the ongoing COVID‐19 pandemic; 283 patients referred to our acute heart failure services over the study period were included on the basis of typical symptoms, raised BNP, and echocardiogram. There was a substantial but statistically non‐significant drop in referrals with 164 referred in the 8 weeks before the first UK death due to COVID‐19 on 2 March 2020 (BC), compared with 119 referred after (AC) in the subsequent 8 weeks, representing a 27% reduction overall (*P* = 0.06). The 30 day case fatality rate was increased from 11% in the BC group compared with 21% in the AC group (risk ratio = 1.9, 95% confidence interval 1.09–3.3). Age, gender, length of stay, left ventricular ejection fraction, and N‐terminal pro‐brain natriuretic peptide were similar between the groups. Admission creatinine, age, and AC cohort status were found to be univariable predictors of mortality. On multivariate Cox regression analysis, only age (hazard ratio 1.04, *P* = 0.03) and AC cohort status (hazard ratio 2.1, *P* = 0.017) remained significant predictors of mortality. On sensitivity analysis, this increased mortality was driven by COVID‐19 positive status.

**Conclusions:**

There was a reduction in referral of patients with acute heart failure with significant increase in mortality in the 8 weeks following the first reported UK death due to COVID‐19. The observation of increased mortality does not appear related to a change in population in terms of demographics, left ventricular ejection fraction, or N‐terminal pro‐brain natriuretic peptide. The observed increased mortality appears to be related to the coexistence of COVID19 infection with acute heart failure. The study highlights the need for widespread preventative and shielding measures particularly in this group of patients especially in the light of the second wave. Longer follow‐up with inclusion of data from other centres and community heart failure services will be needed.

## Introduction

In response to the COVID‐19 pandemic, healthcare services worldwide have introduced unprecedented measures aimed to rationalize resources, including postponement of non‐urgent hospital appointment and elective procedures, and transition to remote consultations to minimize hospital footfall. Recent reports have shown a decline in hospitalization for emergency cardiac conditions such as acute myocardial infarction and heart failure (HF).^1^, ^2^, ^3^ Although recent studies have described the impact of COVID‐19 on HF, demonstrating lower hospitalization and higher mortality, there are few discrepancies and the data are limited especially from the UK.^4^, ^5^, ^6^ In the UK, HF affects 920 000 people, and carries significant mortality, with 1 year survival rates estimated at 81%.^7^, ^8^, ^9^ The British Society for Heart Failure recommends that symptomatic inpatients with new or known HF should be referred to the specialist HF team for assessment and management.^10^

## Methods

We examined inpatient referrals with suspected acute HF to the cardiology team in a large NHS hospital in the south‐west of the UK covering a catchment population of approximately half a million people, over a period of 16 weeks spanning the ongoing COVID‐19 pandemic. Inclusion criteria were BNP levels >400 pg/mL and/or characteristic signs and symptoms of HF^8^, ^11^ alongside echocardiography.

The date of the first coronavirus death in England, 2 March 2020, was used as cut‐off to define two equally sized groups: ‘before COVID‐19’ (BC; 7 January to 2 March 2020, 8 weeks) and ‘after COVID‐19’ (AC; 3 March to 27 April 2020, 8 weeks).^12^

Data for age, gender, N‐terminal pro‐brain natriuretic peptide (NT‐proBNP), echocardiography results, and length of stay were collated. Additionally, the Charlson Comorbidity Index was calculated for each patient on review of electronic notes. Each patient was followed up for 30 days for all‐cause mortality.

Statistical analysis was performed to assess for differences in baseline demographics, as well as type and severity of HF between groups. Categorical variables were presented as absolute numbers, percentages, and risk ratio with 95% confidence interval and compared by the *χ* ^2^ test. Continuous variables were presented as mean and standard deviation and compared by Student's *t*‐test. The association of time variables to mortality was assessed using Kaplan–Meier curves and the log‐rank test. Univariable and multivariable associations of risk covariates with mortality were assessed using Cox proportional hazard regression analyses. An event‐per‐variable ratio of 10 was used, and hence, only variables with *P* < 0.1 in univariable analyses were used in multivariable model. Probability values were two sided, and values of *P* < 0.05 were considered significant. All analyses were conducted using SPSS Statistics (SPSS, IBM Corp, USA, V24) software. The study was registered as a service evaluation audit, and in view of the observational nature of the study, formal ethical approval was not deemed necessary.

## Results

A total of 305 patients were referred to our acute HF services over the study period. All patients referred were reviewed by a consultant cardiologist with subspecialization interest in HF. Based on the consultant review, only those felt to have features suggestive of acute HF who were subsequently managed under the care of a cardiologist were included. This totalled 283 patients (54% male, 81 ± 10 years) in the final analysis.

### Referral pattern and baseline demographics

On review of the referral volume, we observed a substantial, though statistically non‐significant, drop with 164 patients referred before the first UK death due to COVID‐19 (BC), compared with 119 patients referred after (AC), representing a 27% reduction overall (*P* = 0.06).

There was no significant difference in baseline characteristics between the BC and AC groups in terms of age (mean 82 ± 10 vs. 80 ± 11 years, respectively, *P* = 0.12) or gender (female 49% vs. 41%, respectively, *P* = 0.2). Length of stay did not significantly differ between the BC and AC groups (14 vs. 13 days, respectively, *P* = 0.30). There was no difference in Charlson Comorbidity Index comparing the BC and AC groups.

Detailed results on demographics and type of HF are shown in *Table* *1*.

**Table 1:** Demographics and co‐morbidities of study patients according to group

	Before COVID‐19 (BC, *n* = 164)	After COVID‐19 (AC, *n* = 119)	*P*‐value*
Age (years)	82 ± 10	80 ± 11	0.12
Sex (female, %)	49	41	0.2
Previous MI (%)	25	23	0.73
Known HF (%)	69	71	0.68
CCI	8 ± 3	8 ± 3	0.77
Hb (g/dl)	119 ± 23	122 ± 23	0.24
Creatinine (mg/dL)	120 ± 61	131 ± 72	0.16
Diabetes (%)	27	44	0.002
LVEF (%)	45 ± 10	43 ± 11	0.22
NT‐proBNP (pg/ml)	4495	4784	0.377

CCI, Charlson Comorbidity Index; HF, heart failure; LVEF, left ventricular ejection fraction; MI, myocardial infarction.

*P‐value < 0.05 denotes significance.

### Classification and severity of heart failure

Comparing patients before the first UK COVID‐19 death with after, patients were noted to have similar left ventricular ejection fraction, 45 ± 10% vs. 43 ± 11%, *P* = 0.22, with similar proportions of patients with severe left ventricular systolic dysfunction [36% (55/164) vs. 41% (49/119), respectively, *P* = 0.21]. There was a higher median NT‐proBNP level in patients admitted following the first UK COVID‐19 death (AC) compared with before (BC), but this did not reach statistical significance (4784 vs. 4495 pg/mL, respectively, *P* = 0.37).

### Mortality

**Figure 1:**
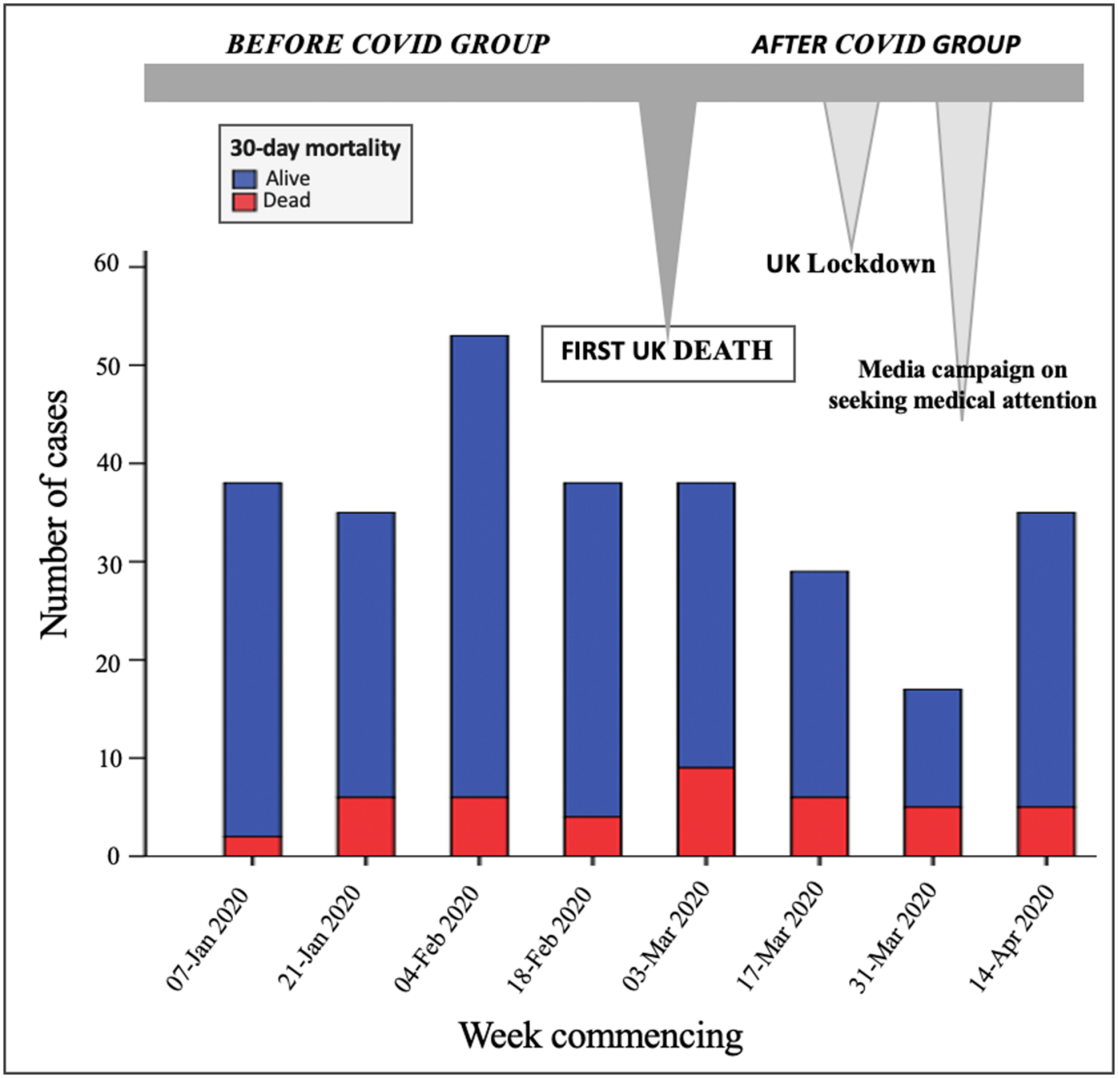
Biweekly referral pattern, with case fatality rate spanning the 16 weeks before and after the first UK death from COVID‐19.

**Figure 2:**
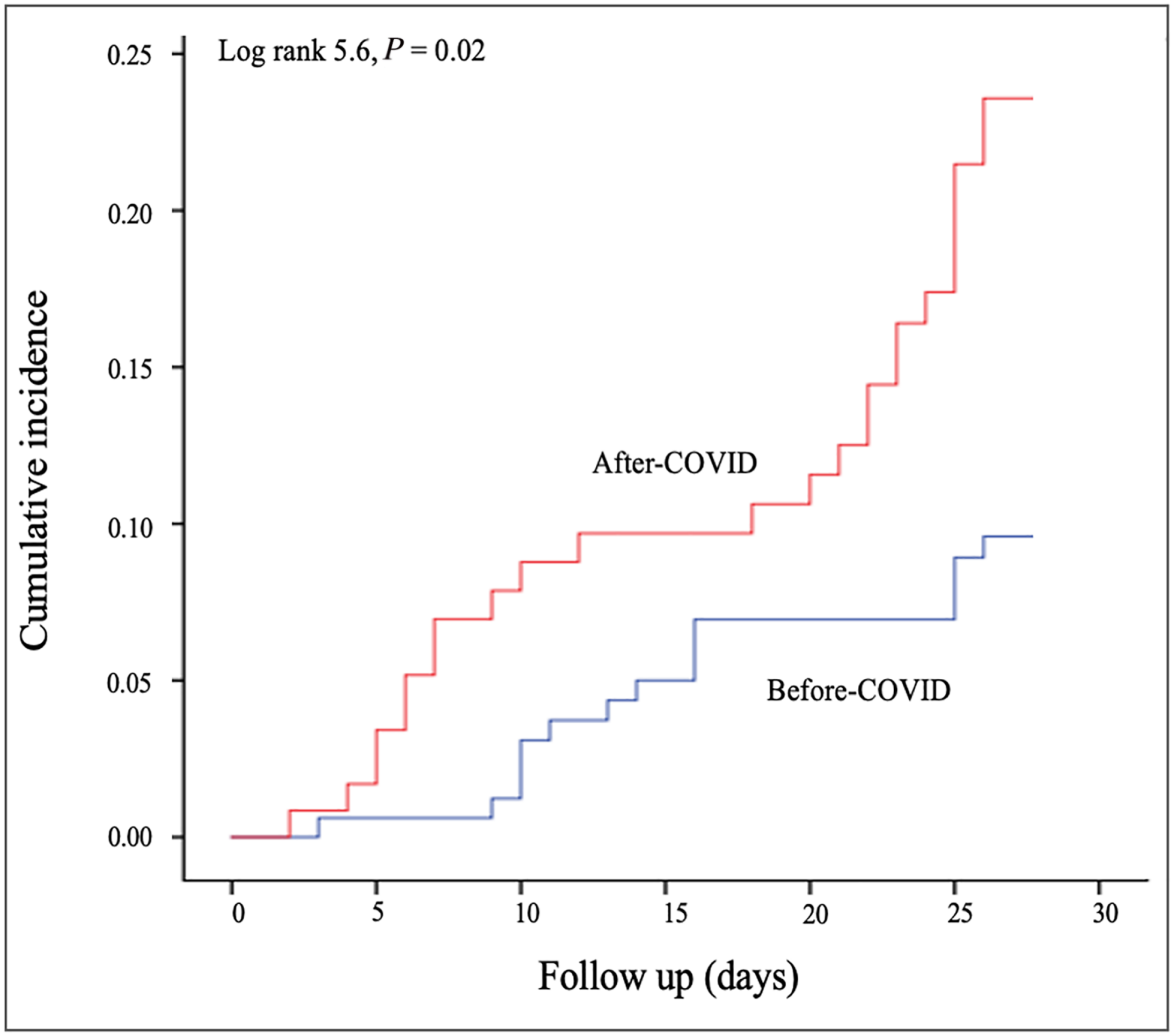
Kaplan–Meier curve demonstrating significant increase in mortality in cohort of patients following the first reported UK death from COVID‐19.

Short‐term (30 day) case mortality was substantially increased to 21% (25/119) in the AC group compared with 11% (18/164) in the BC group (risk ratio = 1.9, 95% confidence interval 1.09–3.3). Biweekly referrals with mortality are shown in *Figure* *1*. *Figure* *2* demonstrates Kaplan–Meier curve analysis showing significant association between the time of presentation and mortality (log rank 5.6, *P* = 0.017).

Univariate and multivariate analysis was performed for the whole cohort using age, gender, admission haemoglobin, admission creatinine, COVID‐19 status, left ventricular ejection fraction, BNP, and group (BC or AC) to look at the predictors of mortality. Only admission creatinine, age, and AC cohort status were found to be univariable predictors of mortality. On multivariate Cox regression analysis, only age (hazard ratio 1.04, *P* = 0.03) and AC cohort status (hazard ratio 2.1, *P* = 0.017) remained significant predictors of mortality. On further analysis of the effect of age on mortality, in the group <80 years, the mortality was 6.3% in BC vs. 13.9% in the AC (log rank 3.7, *P* = 0.05), and in the group ≥80 years, the mortality was 18% in BC vs. 23.2% in the AC (log rank 2.6, *P* = 0.11).

### COVID‐19 status

COVID‐19 testing was carried out in 117 patients, of whom 11 (9.4%) had a positive result. Relating this to short‐term mortality, 6/11 (54%) of COVID‐19‐positive patients died within 30 days vs. 27/106 (25%) of COVID‐19‐negative patients (*P* = 0.07).

On sensitivity analysis, excluding the COVID‐19‐positive patients, short‐term (30 day) case mortality was still higher in the AC group (18.7%, 21/112) compared with the BC group (10%, 16/160), but this became statistically non‐significant (*P* = 0.05).

## Discussion

This study examined the short‐term mortality in hospitalized patients with acute HF in relation to the COVID‐19 pandemic.

We identify a number of important points.

There was a substantial reduction in referral volume to the acute HF service during the COVID‐19 pandemic with an approximately 30% decline in inpatient referrals to cardiology with suspected acute HF after the first UK COVID‐19 death. As reported by Cox *et al*., the reasons for decreased rates of hospitalization during the pandemic are multifactorial.^5^ We suggest that, as reported in other cardiac emergencies, the drop in admissions for acute HF may reflect public concern regarding social distancing during national lockdown and anxiety regarding hospital attendance alongside primary care‐led avoidance of hospital referral. Indeed, this is supported by the drop in referrals being most pronounced at the time of UK lockdown measures and subsequently increasing slightly following media reports encouraging patients to seek medical attention if needed, as indicated in *Figure* *1*.

The major finding of this study is a significant increase in short‐term mortality with an almost two‐fold in case fatality rate. On crude comparisons between the groups by demographics, co‐morbidity index, and length of stay, there was no significant difference between the groups. Although there was no significant difference in age between the BC and AC groups, we demonstrate that age appears to be an independent predictor of mortality. Furthermore, interestingly, we detected a more pronounced, almost statistically significant, increase in mortality in AC vs. BC in the younger (<80 years) age group compared with the same comparison in the older group. Previous studies have identified age as a powerful predictor of mortality in HF.^13^, ^14^ Our study findings are in line with another UK study by Cannatà *et al*. highlighting a reduction in HF hospitalization in two hospitals in London and significantly increased rates of in‐hospital mortality during the COVID‐19 pandemic.^15^ This finding is particularly interesting as London was a high endemic zone compared with Bristol, which had a low case volume during the first wave of COVID‐19. In contrast to the UK, a German study by Bollmann *et al*. showed relatively lower mortality rates of 7%, compared with our study findings.^6^ Another Danish study noted reduced admissions with HF during the pandemic, although it failed to demonstrate a mortality impact.^16^ Studies in other countries have therefore not reported significantly increased mortality as a result of HF in the COVID‐19 pandemic. Finally, we noted a study by Rey *et al*., which looked at 3080 patients with confirmed COVID‐19, reporting significant incidence of acute HF amongst COVID‐19 patients, with high reported associated mortality of 20.5%.^17^

In terms of HF parameters, left ventricular ejection fraction, proportion of those with severe left ventricular dysfunction and NT‐proBNP were similar between the groups suggesting a similar clinical picture on admission. It is possible that the numbers examined are insufficient to demonstrate a more severe clinical picture and that patients with milder symptoms were not seeking medical attention or referral to hospital was being deferred by primary care.

Arguably, the most important aspect demonstrated by this study is that although patients appear to be well balanced in most aspects, the exclusion of positive COVID‐19 patients from the analysis rendered the mortality difference non‐significant. This suggests that the coexistence of acute HF and confirmed COVID‐19 appears to confer a significant poorer prognosis.

This is a novel finding that would ideally be subject to further validation in larger groups but underlines the need for preventative measures to avoid infection particularly to patients with chronic HF. Longer follow‐up and, given the reported striking regional disparity in cases, inclusion of data from other centres as well as data from the community HF service would be needed to fully evaluate this interaction and understand the true implications of the pandemic on emergency cardiac presentations.

### Limitations

- This was a registry and therefore suffers the inherent biases of real‐world observational study with variations in available data and management. However, we feel that this represents important novel data upon which to build.
- We compared patients before and after the first UK death due to COVID‐19. We recognize that there may be seasonal variability in acute HF admissions. However, as the acute HF referral pathway at our institution was implemented in May 2019, it was not possible to make a comparison with previous years.
- We recognize that not all patients in the AC group were tested for COVID‐19. As the pandemic developed, patients were routinely tested on admission to hospital; however, earlier, patients tested were those with symptoms of COVID‐19 or positive contacts. We acknowledge that there are a proportion of untested patients who may have been COVID‐19 positive. Moreover, the symptoms of COVID‐19 and acute HF overlap, and hence, clear distinction between the two may have been missed. The study by Rey *et al*. demonstrated a high prevalence of acute HF in COVID‐19‐positive patients with significantly increased mortality.^17^
- The recorded cause of death was not reported in this study. We recognize that this would have provided additional insight, particularly in those undergoing post‐mortem examinations.
- The study was performed at a district general hospital, and some patients were nursed on mixed cardiology/general medicine wards under the care of a consultant cardiologist. As a result, sensitivity analysis comparing outcome of patients according to type of ward was not possible.

## Conclusions

This study demonstrates a reduction in referrals for acute HF since the onset of the UK COVID‐19 crisis with a significant increase in short‐term mortality after the first UK death. Importantly, our data demonstrate the prognostic impact of positive COVID‐19 status on mortality in patients presenting with acute HF.

## Perspective

- This study highlights the need for widespread preventative and shielding measures particularly in this group of patients presenting with acute HF especially in the light of second wave.
- Longer follow‐up with inclusion of data from other centres and community HF services will be needed to fully evaluate this effect.
- Moving forward, we propose that future work could investigate whether countermeasures adopted in response to the pandemic, such as routine screening of inpatients for COVID‐19, media adverts highlighting the importance of seeking medical attention, or telemedicine (virtual clinics), have impacted on referral patterns and longer‐term mortality.

## Conflict of interest

None declared.

## Author contributions

All authors take responsibility for all aspects of the reliability and freedom from bias of the data presented and their discussed interpretation.
